# Diagnostic PANoptosis-related genes in acute kidney injury: bioinformatics, machine learning, and validation

**DOI:** 10.1080/07853890.2025.2553930

**Published:** 2025-09-02

**Authors:** Zhen Chen, Xiaogang Wang, Yifan Shao, Kai Wang, Dong Xue

**Affiliations:** ^a^ Departments of Urology, The Third Affiliated Hospital of Soochow University, Changzhou, Jiangsu Province, China; ^b^ Department of Urology, Sir Run Run Hospital, Nanjing Medical University, Nanjing, Jiangsu Province, China

**Keywords:** Acute kidney injury, apoptosis, diagnosis, necroptosis, pyroptosis, immune microenvironment

## Abstract

**Background:**

Acute kidney injury (AKI) is a prevalent and life-threatening condition characterized by abrupt renal function decline and subsequent inflammatory cascades. PANoptosis has emerged as a significant contributor to the pathophysiology of AKI. This research aimed to explore the diagnostic and therapeutic implications of PANoptosis-related genes in AKI.

**Methods:**

Kidney biopsy transcriptomic expression data were obtained from the GEO database. Differentially expressed genes (DEGs) associated with PANoptosis were identified between AKI and controls. WGCNA identified hub PANoptosis-related genes. PANoptosis scores and immune cell infiltration were calculated by ssGSEA. Machine learning algorithms was used to select feature genes. ROC analysis evaluated their diagnostic performance. Drug–gene interactions were explored.

**Results:**

We identified 3460 DEGs between AKI and controls (61 upregulated and 11 downregulated) related to PANoptosis, mainly enriched in cytokine signaling and apoptosis. Eight hub PANoptosis genes were identified. PANoptosis scores were significantly higher in AKI patients (*p* < 0.001). CASP8, CASP4, SFN, FAS, and CASP1 were selected as feature genes, with CASP8 having the highest AUC at 0.850 in the training set. A nomogram combining these genes demonstrated strong predictive power. Furthermore, these genes were related to immune cell infiltration positively and had potential drug associations. Validation in a renal ischemia–reperfusion injury rat model confirmed the upregulation of CASP8 (*p* < 0.01), CASP4 (*p* < 0.001), SFN (*p* < 0.0001), FAS (*p* < 0.01), and CASP1 (*p* < 0.01).

**Conclusions:**

Our study identifies PANoptosis-related genes as potential diagnostic markers and therapeutic targets in AKI, highlighting their role in immune dysregulation in AKI.

## 1. Introduction

Acute kidney injury (AKI) is a common clinical complication characterized by an abrupt decline of kidney function, affecting up to 20% of hospitalized patients and carrying mortality rates of 20–50% or higher in severe cases [1,2]. Managing AKI is a critical niche in healthcare, requiring timely diagnosis, addressing underlying causes, and sometimes necessitating renal replacement therapy [3]. Biomarkers and the assessment of immune infiltration have become invaluable tools in the diagnosis and management of AKI [4,5]. In AKI diagnosis, biomarkers like serum creatinine and blood urea nitrogen are commonly used to assess kidney damage. However, these traditional markers have limitations, including delayed elevation after kidney injury and lack of specificity [6]. Newer biomarkers such as neutrophil gelatinase-associated lipocalin (NGAL), kidney injury molecule-1 (KIM-1), and interleukin-18 (IL-18) can detect kidney injury earlier than traditional markers and offer greater sensitivity and specificity [7]. Meanwhile, evaluating immune infiltration involving immune cells like neutrophils, macrophages, and T cells in kidney tissues is crucial for assessing AKI severity, as increased immune infiltration exacerbates inflammation and kidney damage through pro-inflammatory molecule release and immune pathway activation [8,9]. Despite significant accomplishments in AKI biomarker research and immune infiltration analysis, a notable gap persists in biomarkers related to these processes in AKI [10].

AKI can be caused by a rapid loss of renal tubular epithelial cells and subsequent inflammatory cascades [11]. PANoptosis is a recently discovered cellular mechanism that plays a critical role in regulating cell death and immune responses. It has been identified as a biomarker for the early diagnosis or prognosis of various diseases, such kidney cancer [12], diabetic retinopathy [13], idiopathic pulmonary fibrosis [14], aortic dissection [15], and Alzheimer’s disease [16]. This process involves the activation of a combination of programmed cell death pathways, containing necroptosis, apoptosis, and pyroptosis, and is controlled by a set of PANoptosis-related genes [17,18]. In AKI, PANoptosis has emerged as a significant contributor to the pathophysiology. Primary insults such as nephrotoxic substances and ischemia–reperfusion, along with secondary insults including inflammatory cytokines, can trigger PANoptosis in the kidney, leading to AKI through complex interactions between different cell death pathways [19–21]. Furthermore, PANoptosis is closely intertwined with immune infiltration, as the release of inflammatory signals from dying cells can attract immune cells into the kidney tissues [22]. PANoptosis-related genes such as tumor necrosis factor (TNF) superfamily genes and caspases have been implicated in cell death and inflammation in AKI [23,24]. However, the potential clinical significance of PANoptosis-related genes in serving as biomarkers for AKI and their intricate associations with immune infiltration remain largely unknown. Identification of PANoptosis-related genes can not only provide evidences for the early detection of AKI and assessing AKI severity by clinicians, but also holds potential for the prevention, treatment, and recovery of AKI by regulating PANoptosis.

Combined machine learning approaches in bioinformatics can efficiently process complex biological data and identify potential biomarkers that are difficult to detect with traditional methods. Additionally, it can screen out highly specific biomarkers and build more reliable diagnostic models. Here, a comprehensive bioinformatic analyses was performed based on transcriptomic data obtained from the Gene Expression Omnibus (GEO) database to identify PANoptosis-related feature genes in AKI. We assessed their diagnostic accuracy in different datasets, explored their interactions with immune infiltration, and evaluated potential therapeutic implications. The feature genes identified in this research may enhance the accurate prediction and diagnosis of AKI, offering valuable insights onto therapeutic strategies for management of AKI.

## 2. Methods

### 2.1. Data acquisition and processing

Transcriptional expression profiles were procured from the Gene Expression Omnibus (GEO) repository at https://www.ncbi.nlm.nih.gov/geo/, particularly utilizing GSE30718 as the training cohort and GSE217427 as the validation cohort (Table 1). GEO database allows researchers to download and analyze public datasets for scientific purposes. As this study utilized publicly available data without involving direct collection from human participants, it was exempt from ethical approval requirements under local institutional review board guidelines. Compliance with GEO data access and publication policies also confirms that ethical approval was not required. The Medical Ethics Committee of the Third Affiliated Hospital of Soochow University has reviewed this study and exempted the ethics for usage of public database (approval number: No. 2024CL205-01). For preprocessing, each dataset underwent a mapping process where probes were aligned to corresponding gene identifiers, with those probes lacking a significant signal being excluded. In instances where multiple probes aligned to a single gene, the median expression value among those probes was utilized as the representative value for that gene. Subsequently, normalization of the datasets was executed employing the ‘normalizeBetweenArrays’ functionality within the ‘limma’ R package. Regarding the acquisition of PANoptosis-associated genes, two seminal publications served as the foundation. The first study identified an array of 277 genes, segregated into 242 genes tied to apoptosis, 8 genes linked to necroptosis, and 27 genes pertinent to pyroptosis [25]. Complementing this, the second publication contributed an additional 36 genes related to necroptosis [26]. Merging these two comprehensive gene lists resulted in a consolidated set of 313 genes that are intimately involved in the PANoptosis process.

**Table 1.** Data sources and sample information.

ID	Platform	Sample type	Sample size (AKI:control)
GSE30718	GPL570	AKI and control biopsies	47(28:19)
GSE217427	GPL24676	AKI and control biopsies	44(22:22)

AKI, acute kidney injury.

### 2.2. Identification of DEGs related to PANoptosis

The process of differential gene expression analysis was executed on the GSE30718 dataset utilizing the powerful R package, ‘limma.’ Genes displaying a statistical significance with a *p* value <0.05 were identified as DEGs. To refine our target gene set, we intersected these DEGs with a pre-established list of 313 PANoptosis-related genes, resulting in a subset termed DEG_PANoptosis.

### 2.3. Functional enrichment analysis

The DEG_PANoptosis gene set underwent in-depth functional enrichment analyses utilizing both Gene Ontology (GO) and Kyoto Encyclopedia of Genes and Genomes (KEGG) annotations, facilitated by the R package ‘clusterProfiler.’ To account for multiple testing and ensure the reliability of findings, p-values were corrected employing the Benjamini–Hochberg procedure. Consequently, the functional enrichment outcomes were reported and interpreted based on these adjusted *p* values, providing insights into the biological processes and metabolic pathways potentially modulated by these DEG_PANoptosis. The significance threshold of the adjusted *p* value was set at 0.05.

### 2.4. Weighted gene co-expression network analysis (WGCNA)

We constructed a WGCNA network by employing the ‘WGCNA’ R package on GSE30718 dataset to identify AKI-related gene modules. Using the top 50% most variant genes as input, we identified the optimal soft threshold based on scale-free topology criteria. Weighted adjacency and topological overlap matrices were subsequently derived. Utilizing hierarchical clustering with a 0.25 similarity threshold, we pinpointed modules harboring over 30 genes, uniquely color-coded. Further refinement centered on modules significantly correlating with the phenotype. At the nexus of DEG_PANoptosis and top AKI-correlated modules, PANoptosis-linked hub genes were discerned.

### 2.5. PANoptosis scoring

To assess the PANoptosis score within individual samples from the GSE30718 dataset, we performed a single-sample gene set enrichment analysis (ssGSEA) leveraging the ‘GSVA’ toolkit in R. Subsequently, we divided the AKI patient cohort into two groups, distinguished by their respective PANoptosis scores: a high-score group and a low-score group, utilizing the median score as the cutoff. For visualization purposes, we utilized both the ‘Rtsne’ package and principal component analysis (PCA) to illustrate the distribution of PANoptosis scores among the patients. Furthermore, to delve deeper into the biological underpinnings of the observed score variations, we conducted a Gene Set Enrichment Analysis (GSEA) to identify and compare the enrichment of Gene Ontology (GO) functions and Kyoto Encyclopedia of Genes and Genomes (KEGG) pathways between the high- and low-score patient groups.

### 2.6. Immune cell infiltration evaluation

Utilizing ssGSEA and the ‘GSVA’ package, we evaluated the enrichment scores of 28 immune cell subtypes as reported [27,28]. Gene expression data was transformed into gene set expressions to assess enrichment patterns across samples. Wilcoxon test was employed to compare immune cell abundance between groups. Additionally, we explored the relationship between feature genes and immune cell infiltration via Pearson correlation analysis.

### 2.7. Identification of feature genes

Three robust algorithms, including Random Forest (RF), Least Absolute Shrinkage and Selection Operator (LASSO) regression, and Support Vector Machine-Recursive Feature Elimination (SVM-RFE), were used to identify feature genes from PANoptosis-related hubs. For SVM-RFE, the radial basis kernel in the ‘caret’ package was applied, optimizing parameters within ‘rfeControl’ and ‘rfe’ functions. LASSO analysis, conducted with the ‘glmnet’ package, pinpointed optimal penalty λ for non-zero coefficient genes. Random Forest ranked genes based on Gini coefficients >1 as significant. By intersecting the genes identified by these three algorithms, a refined set of feature genes was obtained. This comprehensive approach ensures robust identification of genes crucial to PANoptosis pathways.

### 2.8. Protein–protein interaction (PPI) network

To explore interactions among feature genes and their functional counterparts, a PPI network was generated leveraging the STRING database (https://cn.string-db.org/) for feature gene analysis.

### 2.9. Nomogram construction and evaluation

A customized nomogram, tailored from discriminatory gene features, was crafted using the ‘rms’ R library. For intuitive understanding, it was visualized *via* the ‘regplot’ approach. Its clinical utility was rigorously evaluated through calibration plots generated by ‘rms’’s ‘calibrate’ function, complemented by decision curve analysis (DCA) visuals crafted with the ‘ggDCA’ package, ensuring precise assessment of diagnostic and treatment decision impacts.

### 2.10. Drug–gene interaction analysis

We leveraged the DGIdb resource to investigate the interplay between drugs and key genes. The intricate network of interactions was then graphically represented using Cytoscape, facilitating insights into drug–gene dynamics.

### 2.11. Animal model

To validate alterations in biomarker genes, a rat model of renal ischemia–reperfusion injury (RIRI) was devised as described in our previous work [29]. Sixteen male, 8-week-old, healthy rats (sourced from Hangsi-Bio, Hangzhou, China) were randomized into Sham and RIRI cohorts. Under 2% isoflurane anesthesia, Sham rats underwent laparotomy with subsequent closure after 45 min. RIRI rats, however, endured left nephrectomy, with clamping of the right renal artery for 45 min, followed by clamp release, abdominal closure, and 24 h of reperfusion. Subsequently, both groups were anesthetized, and right kidneys were procured for in-depth analysis, aiming to elucidate the effects of RIRI on gene expression. YFS conducted of the experiment in August 2023 and XGW assessed the outcome and analyzed the data. This research was approved by the Institutional Animal Care and Use Committee of Soochow University (approval number: No. 2022CL038-01), performed according to the National Institutes of Health guidelines, and carried out in compliance with the ARRIVE guidelines.

### 2.12. Quantitative real-time polymerase chain reaction (qRT-PCR)

RNA from kidney tissues of Sham and RIRI rats was extracted with a kit (Nanjing Vazyme, Jiangsu, China). cDNA was synthesized, followed by qPCR using ChamQ SYBR mix (Nanjing Vazyme). GAPDH served as a reference. Triplicate reactions were performed. Relative gene expression was analyzed by the 2^–ΔΔ^ Ct method. Primer sequences are listed in Table S1.

### 2.13. Western blot

Western blot was conducted as described in the previous literature [29]. Briefly, total protein was extracted from kidney tissues of Sham and RIRI rats by using RIPA buffer. After determining the concentration of protein samples, all protein samples were loaded onto 10% sodium dodecyl‐sulfate polyacrylamide gel electrophoresis and transferred to polyvinylidene fluoride membranes. After blocking with 5% milk, the membranes were incubated with primary antibody. Then, the membranes were washed and incubated with the secondary antibody and exposed ECL Kit (Thermo Scientific, USA). The antibodies are listed as follows: anti-CASP8 (#13423-1-AP, 1:500, Proteintech, China), anti-CASP4 (#11856-1-AP, 1:500, Proteintech), anti-SFN (#R381409, 1:500, Zenbio, China), anti-FAS (#YT1676, 1:500, Immunoway, USA), anti-CASP1 (#A0964, 1:1000, Abclone, China), and anti β-actin (#66009-1-Ig, 1:5000, Proteintech).

### 2.14. Statistical analysis

Statistical evaluations employed R (v4.3.0), encompassing PCA visualized by ‘FactoMineR’ and ‘factoextra’, heatmaps crafted with ‘pheatmap’, Venn diagrams by ‘ggvenn’, and ROC curves plotted through ‘pROC’. Gene correlations were visualized with ‘ggpairs’, while ‘ggplot2’ and ‘plot’ facilitated diverse visualizations. Continuous data were compared between groups using the Wilcox test. qRT-PCR data analysis utilized GraphPad Prism 8.0 for graphing and an unpaired *t* test to compare groups, deeming *p* < 0.05 as statistically significant. Pearson correlation analysis assessed gene relationships.

## 3. Results

### 3.1. Identification and characterization of DEG_PANoptosis between AKI and controls

**Figure 1.**
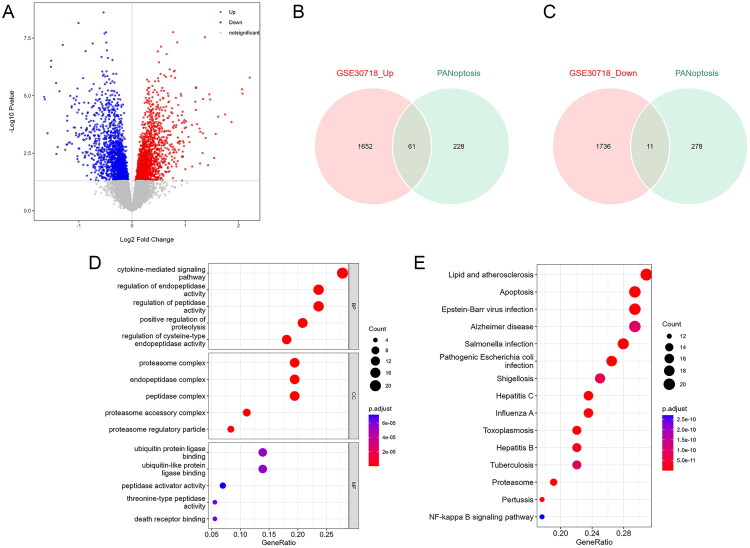
Identification and enrichment analysis of differentially expressed genes related to PANoptosis (DEG_PANoptosis) in acute kidney injury (AKI). (A) A volcano plot displays 3460 DEGs between AKI and non-AKI controls from the GSE30718 dataset. (B,C) The DEGs were intersected with 313 genes related to PANoptosis, resulting in 61 upregulated DEG_PANoptosis and 11 downregulated DEG_PANoptosis. (D) GO enrichment analysis of the DEG_PANoptosis. BP, biological processes; CC, cellular component; MF, molecular function. (E) KEGG pathway enrichment analysis of the DEG_PANoptosis.

We collected gene expression data from kidney biopsies in the GSE30718 dataset to identify DEG_PANoptosis that distinguish AKI from non-AKI controls. A total of 3460 DEGs were identified (Figure 1A; Table S2). Further analysis revealed an intersection between the DEGs and PANoptosis-related genes, generating 61 upregulated and 11 downregulated DEG_PANoptosis (Figure 1B and 1C). Results of GO enrichment indicated that the 72 DEG_PANoptosis were mainly related to several biological processes and molecular functions, including ‘Cytokine-mediated signaling pathway’, ‘Regulation of endopeptidase activity’, ‘Ubiquitin–protein ligase binding’, and ‘Death receptor binding’ (Figure 1D; Table S3). KEGG analysis revealed that the DEG_PANoptosis were significantly associated with ‘Apoptosis’ and ‘NF-kappa B signaling pathway’ (Figure 1E; Table S4).

### 3.2. Identification of hub genes related to PANoptosis in AKI

**Figure 2.**
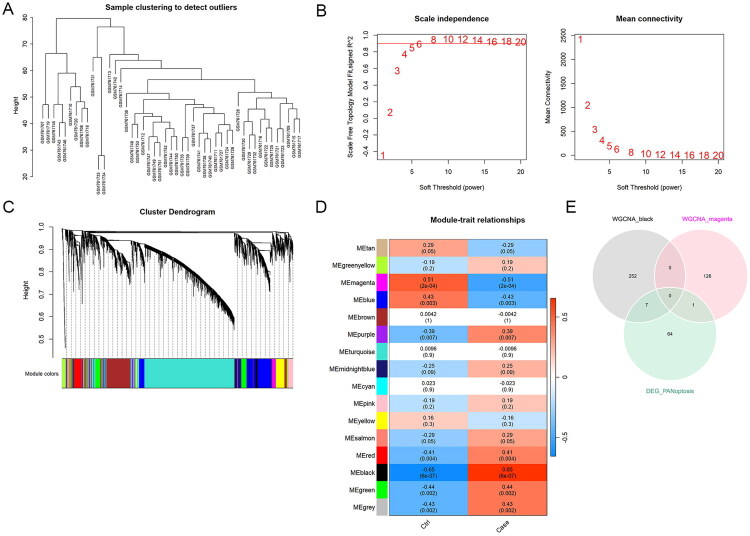
Gene co-expression analysis using WGCNA. (A) Initial sample clustering for the GSE30718 dataset. No outliers were detected. (B) Determination of the soft threshold power (β) to establish a scale-free topology and optimal connectivity. β was set to 5. (C) A hierarchical clustering dendrogram shows the categorization of genes into 15 distinct modules. Each color below the dendrogram represents a module. (D) A heatmap displays module-trait relationships. The color intensity indicates the strength of the relationship between modules and traits. (E) The 72 DEG_PANoptosis were intersected with the genes within the black and magenta modules, resulting in eight hub genes.

To identify core modules related to AKI, we conducted WGCNA on the GSE30718 dataset. After initial sample clustering, we did not detect any outlier (Figure 2A). Optimal scale-free connectivity was established with β = 5 (Figure 2B), yielding 15 gene modules through hierarchical clustering (Figure 2C). The black and magenta modules exhibited the highest correlation (*R* = 0.65 and 0.51, respectively) with AKI (Figure 2D). By intersecting the genes within the two modules and 72 DEG_PANoptosis, we identified eight hub genes, including CASP8, SFN, CASP4, TNFRSF1A, MCL1, LMNB1, CASP1, and FAS, for further analysis (Figure 2E).

### 3.3. PANoptosis involvement and hub gene expression in AKI

**Figure 3.**
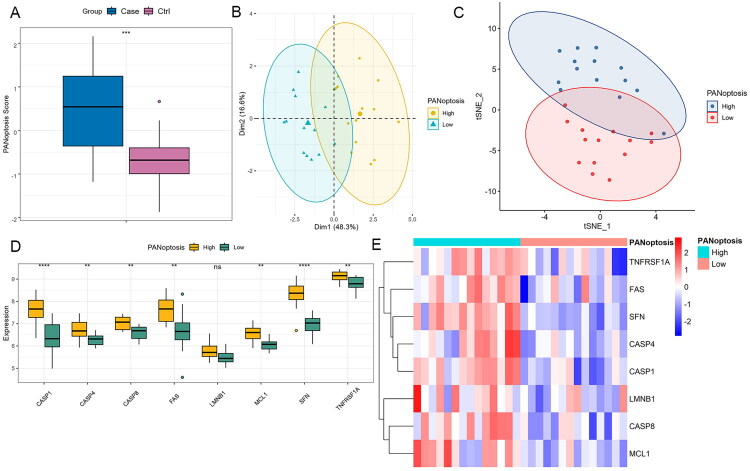
PANoptosis scores and hub gene expression in the GSE30718 dataset. (A) a box plot illustrates the difference in PANoptosis scores between the AKI patients and the controls in the GSE30718 dataset. (B, C) AKI patients were divided into high and low-score subgroups based on the median PANoptosis score. Principal component analysis (PCA) analysis (B) and t-distributed stochastic neighbor embedding (tSNE) visualization (C) demonstrate the separation of the two subgroups. (D,E) Box plot and heatmap of hub gene expression between high and low-score subgroups.

To assess PANoptosis involvement in AKI, we computed sample-specific scores in the GSE30718 cohort using ssGSEA with eight hub genes. Scores were significantly elevated in AKI patients versus controls (Figure 3A). Stratification by median PANoptosis scores divided patients into high/low subgroups. Both PCA and t-SNE dimensionality reduction techniques showed clear separation between subgroups (Figure 3B,C). Except for LMNB1, all the other seven hub genes were significantly upregulated in high-score patients compared to low-score patients (Figure 3D and 3E). These data suggest that PANoptosis may play a crucial role in the pathogenesis of AKI and that the expression levels of the hub genes could serve as potential biomarkers for the disease.

### 3.4. Immune infiltration and hub gene correlation analysis in AKI

**Figure 4.**
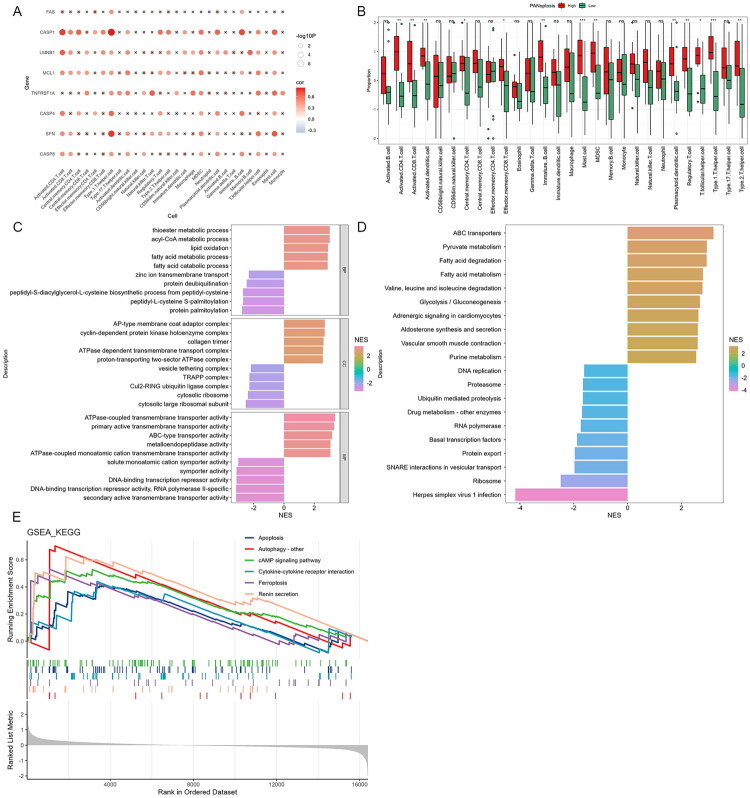
Correlation and enrichment analyses of hub genes with immune cells and pathways in different patient subgroups. (A) A dot plot displays the correlation between different immune cells with hub genes. (C,D) GO and KEGG enrichment analyses using GSEA. NES, normalized enrichment score. An NES greater than 0 suggests activation of the pathway in the high-score subgroup, while an NES less than 0 indicates suppression of the pathway in the same group. (E) GSEA enrichment plot displaying the distribution and significance of key pathways.

To investigate the relationship between immune response and PANoptosis in AKI patients, we analyzed the correlation between hub genes and immune cells. The results demonstrated a varying degree of positive correlation between the expression levels of hub genes and the abundance of immune cell types. (Figure 4A). Among the 28 immune cell types analyzed, all of them exhibited upregulated trends in high-score patients. Notably, 13 of these immune cell types showed a significant increase in high-score patients when compared to low-score patients, including activated CD4 T cells, activated CD8 T cells, activated dendritic cells, central memory CD4 T cells, effector memory CD8 T cells, immature B cells, mast cells, myeloid-derived suppressor cells (MDSCs), plasmacytoid dendritic cells, regulatory T cells (Tregs), T follicular helper cells (Tfh), type 1 T helper cells (Th1), and Th2 cells (Figure 4B). GSEA further revealed that the most significant GO terms with positive enrichment in the high-score group included ‘acyl-CoA metabolic process,’ ‘AP-type membrane coat adaptor complex’, and ‘ATPase-coupled transmembrane transporter activity’. On the contrary, terms like ‘zinc ion transmembrane transport’ and ‘DNA binding transcription factor activity’ were among those significantly suppressed (Figure 4C; Table S5). In addition, pathways such as ‘ATP-binding cassette (ABC) transporters’ and ‘Fatty acid degradation’ were notably activated, whereas pathways like ‘Ribosome’ and ‘DNA replication’ were notably suppressed in the high-score group (Figure 4D; Table S6). GSEA enrichment plots revealed marked activation of apoptosis, autophagy, and cytokine–cytokine receptor pathways in the high-score subgroup (Figure 4E). These results suggest that in AKI patients with a high PANoptosis score, there is an enhanced immune response, potentially contributing to disease progression through significant signaling pathways such as apoptosis.

### 3.5. Identification of feature genes related to PANoptosis in AKI

**Figure 5.**
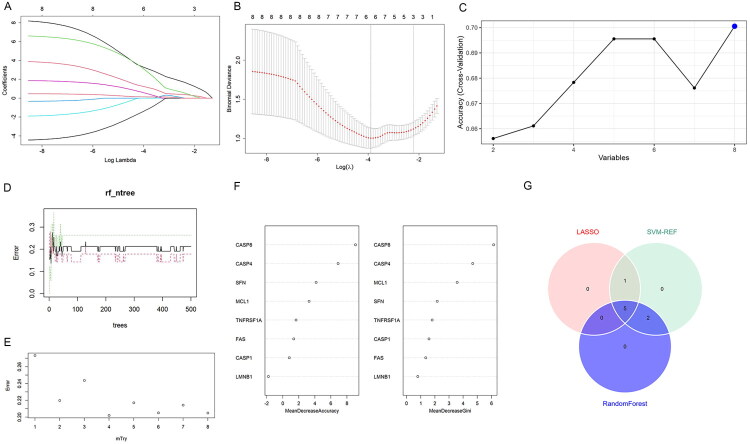
Identification of feature genes. (A) The coefficients of genes in a LASSO regression model. The optimal lambda value identifies six genes with non-zero coefficients. (B) The tuning of the lambda parameter in the LASSO regression. The vertical lines represent the error at different lambda values, with the red dotted line representing the optimal lambda with minimum error. (C) The accuracy of the support vector machine recursive feature elimination (SVM–RFE) model. A peak at eight feature genes indicates the highest model accuracy. (D) The error rate in a random Forest (RF) model stabilizes when the number of trees exceeds 300. (E) The error rate varies with different values of the mtry parameter in the RF model, with the lowest error corresponding to an mtry of 4. (F) The importance of different genes was determined by the mean decrease in accuracy and gini coefficient in the RF model. Genes with a Gini coefficient greater than 1 were selected as feature genes. (G) A venn diagram represents the intersection of feature genes identified by the LASSO, SVM-RFE, and RF algorithms, resulting in five feature genes relevant to AKI.

To determine AKI-associated feature genes, three machine learning approaches were applied to screen candidates from the initial eight hub genes. LASSO regression analysis with optimized lambda parameters identified six genes through non-zero coefficient selection (Figure 5A,B). The SVM–RFE algorithm demonstrated superior performance by achieving the highest accuracy while retaining all eight candidate genes (Figure 5C). For the random forest model, stability in error rates emerged when utilizing over 300 decision trees (Figure 5D), with optimal classification performance occurring at mtry = 4 (Figure 5E). Seven genes with a Gini coefficient greater than 1 were selected as feature genes (Figure 5F). The intersection of genes identified by the three algorithms yielded five feature genes, including CASP8, CASP4, SFN, FAS, and CASP1 (Figure 5G). A PPI network showed that these feature genes had multiple connections with other proteins (Figure S1A). In the GSE30718 dataset, the feature genes showed significant positive correlations with each other (Figure S1B). These data suggest that the identified feature genes play a significant interconnected role in the pathogenesis of AKI.

### 3.6. Evaluation of feature genes for diagnosing AKI

**Figure 6.**
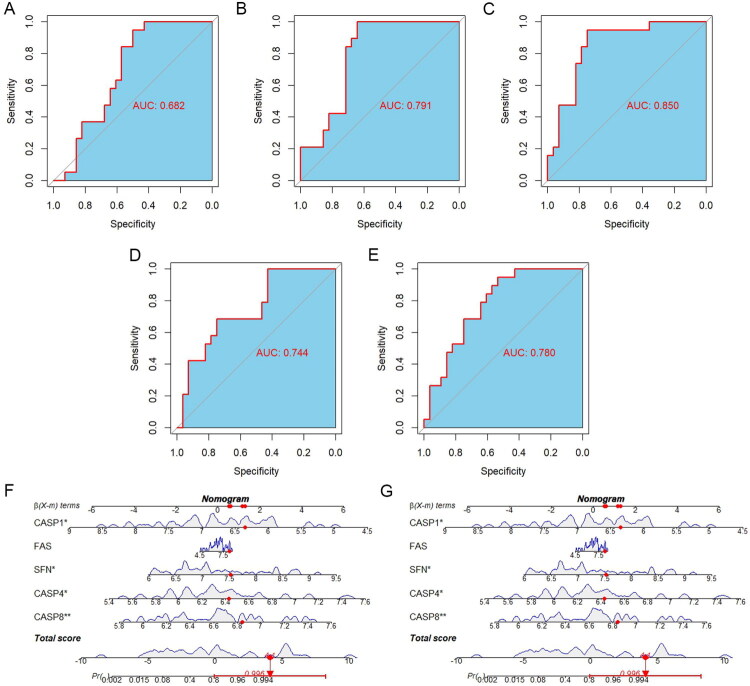
Evaluation of feature genes for predicting AKI. (A–E) Receiver operating characteristic (ROC) curves for the evaluation of feature genes in diagnosing AKI in the GSE30718 dataset. (F) A nomogram was constructed based on the expression of feature genes. Each gene contributes a score on the scale, providing a total score associated with the probability of AKI. (G) A calibration curve comparing the predicted probabilities of AKI against the observed probabilities. (H) Decision curve analysis (DCA) graphs comparing the net benefit of using the composite model versus individual feature genes for predicting AKI.

We evaluated the diagnostic accuracy of each feature gene by calculating the Area Under the ROC curve (AUC). In the training set GSE30718, CASP8 had the highest AUC at 0.850, followed by CASP4 at 0.791, SFN at 0.780, FAS at 0.744, and CASP1 at 0.682, all showing good diagnostic performance (Figure 6A–E). However, in the validation dataset GSE217427, we observed lower AUC values compared to the training set, ranging from 0.568 to 0.793. CASP4 demonstrated the highest AUC among the biomarkers (Figure S1C–G). This suggests a variance in the performance of these genes, possible due to differences in sample populations or data collection methods. Feature genes exhibited significant upregulation or a trend towards upregulation in AKI patients compared to controls in both training and validation datasets (Figure S2A and S2B). Pearson correlation analysis revealed a significant positive correlation between each gene and the PANapoptosis score, further substantiating their role in AKI pathogenesis (Figure S2C). This suggests that higher expression levels of these feature genes are associated with increased PANapoptosis signaling in AKI, highlighting their potential as diagnostic biomarkers.

To evaluate the clinical utility of feature genes in AKI risk prediction, we developed a nomogram incorporating gene expression profiles (Figure 6F). Calibration analysis revealed strong concordance between model-predicted risks and actual clinical outcomes (Figure 6G). Decision curve analysis further confirmed that the integrated model provided greater clinical net benefit compared to single-gene predictors (Figure 6H). These multimodal validation approaches collectively indicate that the combinatorial gene signature offers robust diagnostic accuracy for AKI identification.

### 3.7. Correlation with immune microenvironment and therapeutic implications of feature genes in AKI patients

**Figure 7.**
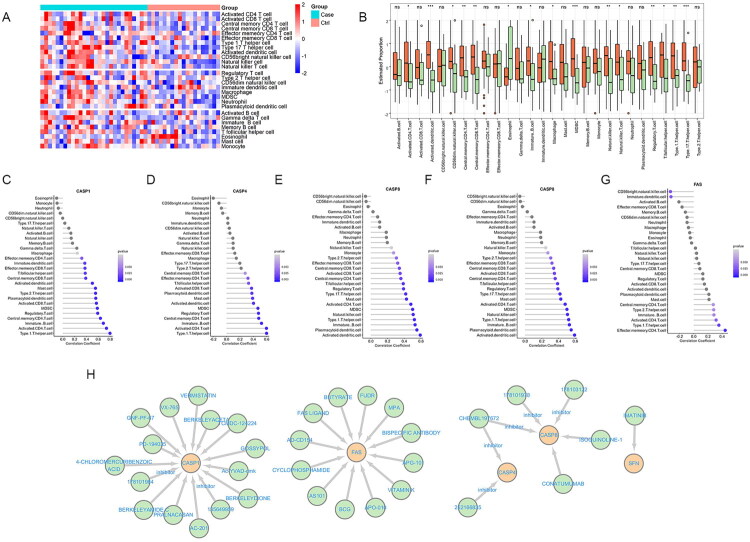
Immune cell subtype distribution and gene–drug interactions in AKI. (A) A heatmap demonstrates the distribution of immune cell subtypes across various samples within the GSE30718 dataset. (B) Boxplots illustrate the comparative abundance of immune cell subtypes between AKI cases and controls. (C–G) Lollipop charts show the correlation coefficients between the abundance of immune cell subtypes and the expression levels of five feature genes. (H) Gene–drug interactions.

To compare immune microenvironment differences between AKI patients and controls, we analyzed immune cell abundance in the GSE30718 dataset. Apart from eosinophils, we noted an upregulation trend in all other immune cell types among AKI patients, with significant differences observed in 15 of these immune cell types when compared to the control group (Figure 7A and 7B). The five feature genes demonstrated positive associations with various immune cell types, with no significant negative correlations detected. Specifically, CASP1, CASP4, and SFN showed strong positive correlations Th1 cells. CASP8 was closely correlated with activated dendritic cells, and FAS was significantly associated with effector memory CD4 T cells (Figure 7C–G). To study the therapeutic implications of the feature genes, we conducted an analysis using the DGIdb database. CASP1 was prominent with a substantial number of associated drugs, including VX-765, a targeted caspase activity inhibitor. In contrast, SFN appeared to have a narrower interaction profile, notably with the well-known cancer drug imatinib. FAS also had significant drug associations, while CASP4 and CASP8 showed fewer interactions (Figure 7H; Table S7). This study suggests that the identified feature genes in AKI patients are strongly correlated with certain immune cells and have varying potential for targeted drug therapy.

### 3.8. Validation of feature gene alterations in RIRI rat model

**Figure 8.**
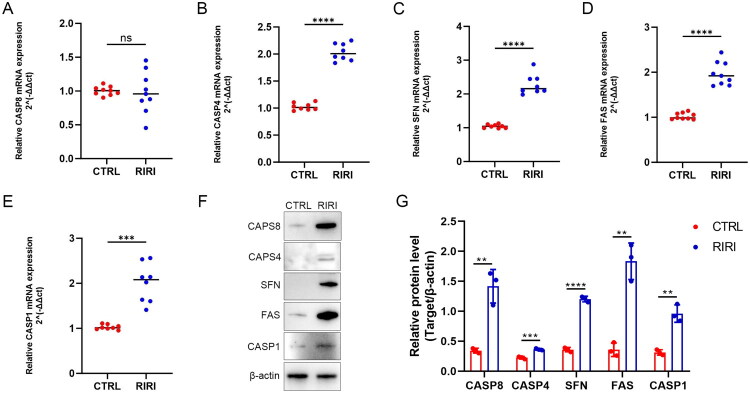
mRNA and protein expression of feature genes in kidney samples from a RIRI rat model. (A–E) qRT-PCR analysis was conducted to determine mRNA levels of CASP8, CASP4, SFN, FAS, and CASP1 in kidney tissues obtained from sham rats and RIRI rats (*n* = 8). (F-G) Western blot analysis was conducted to determine protein levels of CASP8, CASP4, SFN, FAS, and CASP1 in kidney tissues obtained from sham rats and RIRI rats (*n* = 3). Data are presented as mean ± SD. Statistical significance was determined using an unpaired *t* test. **p < 0.01, ***p < 0.001, ****p < 0.0001, ns: non-significant.

Next, we sought to verify feature gene alterations in kidney samples from RIRI rats. Through qRT-PCR analysis, we observed substantial upregulation of CASP4, SFN, FAS, and CASP1 in the RIRI group compared to the Sham group (all *p* < 0.05; Figure 8A–E), except for CASP8, which was not significant. Western blot results showed that these feature gene expressions at the protein level were similar with that at the mRNA level besides CASP8 (Figure 8F–G; Figure S3). These are mostly consistent with what we observed in the bioinformatics analysis.

## 4. Discussion

In this study, we identified and characterized a set of feature genes related to PANoptosis in AKI using comprehensive bioinformatic analyses. These feature genes, including CASP8, CASP4, SFN, FAS, and CASP1, were found to be associated with AKI and displayed strong correlations with immune cell infiltration, suggesting their potential as diagnostic biomarkers. Additionally, their positive associations with specific immune cells and the availability of drugs targeting these genes indicate potential therapeutic implications, highlighting the clinical significance of these findings for improving AKI diagnosis and treatment. The validation results in the RIRI rat model further confirmed the substantial upregulation of CASP4, SFN, FAS, and CASP1, reinforcing their relevance as biomarkers.

Vigorous research in the field of AKI focuses on pathophysiological features, including topics such as oxidative stress, inflammatory responses, apoptosis, necrosis, metabolic dysregulation, and immune responses. However, current researches of PANoptosis in AKI are limited. In this study, CASP1, CASP4, and CASP8 were identified as feature genes associated with PANoptosis in AKI. They were upregulated in AKI patients and showed significant correlations with PANoptosis scores, indicating their potential as diagnostic biomarkers. CASP8 displayed the highest diagnostic accuracy (AUC = 0.850) in the training dataset. While the diagnostic performance varied slightly in the validation dataset, these genes demonstrate strong potential for AKI diagnosis. The caspase family of proteins plays a pivotal role in the complex landscape of cell death pathways, influencing various processes in AKI. CASP1 plays a critical role in regulating pyroptosis, a programmed cell death process characterized by gasdermin D cleavage and membrane pore formation in AKI. Its activation leads to cell swelling, membrane rupture, and death, along with the release of inflammatory factors like IL-18 and IL-1β, *via* the NLRP3 inflammasome, contributing to the inflammatory response and cell death in AKI [30,31]. CASP4, along with CAPS5 and CAPS11, is activated independently of inflammasomes, primarily by lipopolysaccharide binding, and is crucial for cleaving gasdermin D, leading to pyroptosis and associated inflammatory responses in AKI [32,33]. CASP8 serves as a central regulator in the intricate network of cell death pathways. It activates apoptosis by interacting with apoptosis-associated speck-like protein and enhances the apoptotic cascade while inhibiting necroptosis [22,34,35]. CASP8 also plays a role in the crosstalk between autophagy and apoptosis, with its activation affecting the transition between these pathways [36]. Our results may help in understanding these interactions and developing potential therapeutic strategies for AKI.

We also identified SFN and FAS as feature genes associated with immune responses in AKI. Both genes were positively correlated with the PANoptosis score, indicating their involvement in PANoptosis signaling in AKI. Consistent with our findings, researchers have observed elevated SFN levels in AKI patients and mouse models. In human kidney cells and mouse models, knocking down SFN reduced kidney injury markers, mitigated programmed cell death, and suppressed inflammation. SFN also interacts with RIPK3, a key regulator of necroptosis, leading to necroptosis and subsequent inflammation in kidney cells [37]. In AKI, the FAS (Fas cell surface death receptor) system is involved in initiating extrinsic apoptosis, primarily through the binding of FAS ligand (FASL) to the FAS receptor on renal cells. Activation of FAS receptor triggers a cascade of signaling pathways that lead to the activation of initiator caspases (mainly CASP8 and CASP10) and subsequently effector caspases (CASP3, 6, and 7), ultimately inducing apoptosis in renal cells [38–40]. Additionally, FAS can contribute to necroptosis in AKI, leading to the formation of a necroptosome containing RIPK1, RIPK3, and MLKL, ultimately resulting in cell death [41]. Therefore, FAS is implicated in both apoptosis and necroptosis in AKI.

Our results indicate that in AKI patients, there was an increased abundance of various immune cell types, with significant differences observed in 15 immune cell types when compared to the control group, suggesting an enhanced immune response in AKI. Additionally, the feature genes identified in AKI patients were positively correlated with different immune cell types. Notably, CASP1, CASP4, and SFN exhibited strong positive correlations with Th1 cells, while CASP8 was closely associated with activated dendritic cells, and FAS showed a significant correlation with effector memory CD4 T cells. Indeed, immune cells like dendritic cells, monocytes/macrophages, neutrophils, T lymphocytes, and B lymphocytes play roles in causing kidney damage during AKI. Conversely, M2 macrophages and Tregs help suppress inflammation and support tissue repair after AKI [42]. In AKI, T-cell receptor (TCR) engagement contributes to renal injury after ischemia–reperfusion injury, involving both TCR-repertoire-dependent and -independent factors [43]. Blocking FASL reduces renal injury by limiting TNF-producing T-cell infiltration into post-ischemic kidneys. In cisplatin-induced AKI, T cells, especially CD4+ T cells, play a crucial role in renal injury [44]. CD4-deficient mice demonstrate lower mortality and renal impairment compared to wild-type counterparts [45]. Cisplatin-induced AKI pathogenesis involves FASL-mediated apoptosis, triggered by FASL expression in renal tubular cells and infiltrating immune cells, especially T lymphocytes.[46]. Our results emphasize the critical role of immune infiltration and the feature genes in AKI pathogenesis, suggesting their potential as therapeutic targets for mitigating immune responses and tissue damage in AKI.

The feature genes CASP1, CASP4, CASP8, SFN, and FAS in AKI patients showed varying drug associations, with CASP1 and FAS having numerous drug interactions, while CASP4, CASP8, and SFN were notably associated with specific drugs. In the drug–gene network, some compounds are known to be involved in inflammation and PANoptosis. Cyclophosphamide, for instance, can suppress the immune system and is used in immune therapies for autoimmune diseases and organ transplantation [47]. The caspase-1 inhibitor VX-765 emerges as a promising therapeutic candidate for mitigating the progression of diabetic nephropathy, as it effectively suppresses caspase-1-mediated pyroptosis, renal inflammation, and fibrosis in diabetic mice [48]. Vitamin K1 has been identified as an effective inhibitor of ferroptosis, offering a promising strategy for preventing this mode of cell death in conditions like acute tubular necrosis during AKI [49]. These data suggest that targeting the identified feature genes may hold promise for developing novel therapeutic approaches for AKI.

One limitation involves the dependence on bioinformatics and gene expression analyses, which might overlook the dynamic complexity of AKI in clinical contexts. The findings are primarily based on gene expression patterns, and further experimental validation, such as functional assays and clinical studies, is needed to confirm the roles of these identified feature genes and their diagnostic or therapeutic potential in AKI. Additionally, the study relies on publicly available datasets, which may have variations in sample populations and data collection methods, leading to potential biases or discrepancies in the results. More surveys from different countries are still needed. Moreover, while drug associations were explored, the actual efficacy and safety of these drugs in AKI patients were not directly assessed in this study, warranting further investigation and clinical trials. Another limitation is the lack of CASP8 upregulation at mRNA level in the RIRI rat model, possibly due to variations in the experimental conditions or differences in the underlying pathophysiological mechanisms between human AKI and the RIRI rat model. However, the protein level change of CASP8 in rat was consistent with which in human. Finally, more studies with multiple omics methods, such as proteomics and metabolomics, integrate different types of molecular data, which can provide a more comprehensive understanding of the AKI pathophysiology and the role of PANoptosis in AKI.

## 5. Conclusion

This research identified and characterized a set of DEGs associated with PANoptosis in AKI. These genes, including CASP8, CASP4, SFN, FAS, and CASP1, were found to be interconnected and associated with the pathogenesis of AKI. The study demonstrated their potential as diagnostic biomarkers for AKI, with a composite model showing promising accuracy. Besides, the feature genes exhibited correlations with immune cell types and potential therapeutic implications, though further validation and clinical studies are required. Despite the insights gained, it’s important to acknowledge the study’s limitations in terms of relying on bioinformatics analysis and the need for future experimental and clinical investigations to translate these findings into practical applications for AKI management.

## Supplementary Material

Supplemental Material

Figure S3.tif

Author Checklist.pdf

Table S1_7.xlsx

Figure S1.tif

Figure_S2 (1).tif

## Data Availability

All data generated or analysed during this study are included in this published article [and its supplementary information files]. They also can be available from the corresponding author, KW or DX, upon reasonable request.
