# Supplementary material for: Diagnostic PANoptosis-related genes in acute kidney injury: bioinformatics, machine learning, and validation
Source: Ann Med. 2025 Sep 2;57(1):2553930. doi: 10.1080/07853890.2025.2553930 (PMC12406322; doi:10.1080/07853890.2025.2553930)
Supplement: Supplemental Material [file IANN_A_2553930_SM5624.docx]

**Figure S1. Interactions of feature genes and validation of their diagnostic values in the external dataset.** (A) Protein-protein interaction (PPI) network. (B) Pearson correlation analysis of each pair of feature genes. The distribution of the expression of each feature gene in AKI (blue) and controls (yellow) is displayed on the diagonal. The Pearson correlation coefficients and corresponding scatter plots are displayed in the upper right and lower left triangles, respectively. **P < 0.01, ***P < 0.001. (C-G) ROC curves for the evaluation of feature genes in diagnosing AKI in the GSE217427 dataset.

**Figure S2. Comparative expression and correlation analysis of feature genes in AKI.** (A, B) The differential expression of feature genes (CASP1, CASP4, CASP8, FAS, SFN) in AKI patients versus controls in the training dataset GSE30718 and validation dataset GSE217427. *P < 0.05, **P < 0.01, ***P < 0.001, ****P < 0.001, ns: non-significant. (C) Pearson correlation analysis between the PANapoptosis score and the expression levels of the respective feature genes in the GSE30718 dataset. R, correlation coefficient.

**Figure S3. Unprocessed Western blot of Figure 8F.**

**Table S1. qRT-PCR Primers.**

**Table S2. Differentially expressed genes related to AKI identified in the GSE30718 dataset.**

**Table S3. GO enrichment analysis of the 72 DEG_PANoptosis.**

**Table S4. KEGG analysis of the 72 DEG_PANoptosis.**

**Table S5. Significantly enriched and suppressed GO terms in high-score vs. low-score AKI patients as revealed by GSEA.**

**Table S6. Significantly enriched and suppressed KEGG pathways in high-score vs. low-score AKI patients as revealed by GSEA.**

**Table S7. Therapeutic drug associations of feature genes in AKI patients as analyzed using the DGIdb database.**
