# Supplementary figures and images for: Diagnostic PANoptosis-related genes in acute kidney injury: bioinformatics, machine learning, and validation
Source: Ann Med. 2025 Sep 2;57(1):2553930. doi: 10.1080/07853890.2025.2553930 (PMC12406322; doi:10.1080/07853890.2025.2553930)

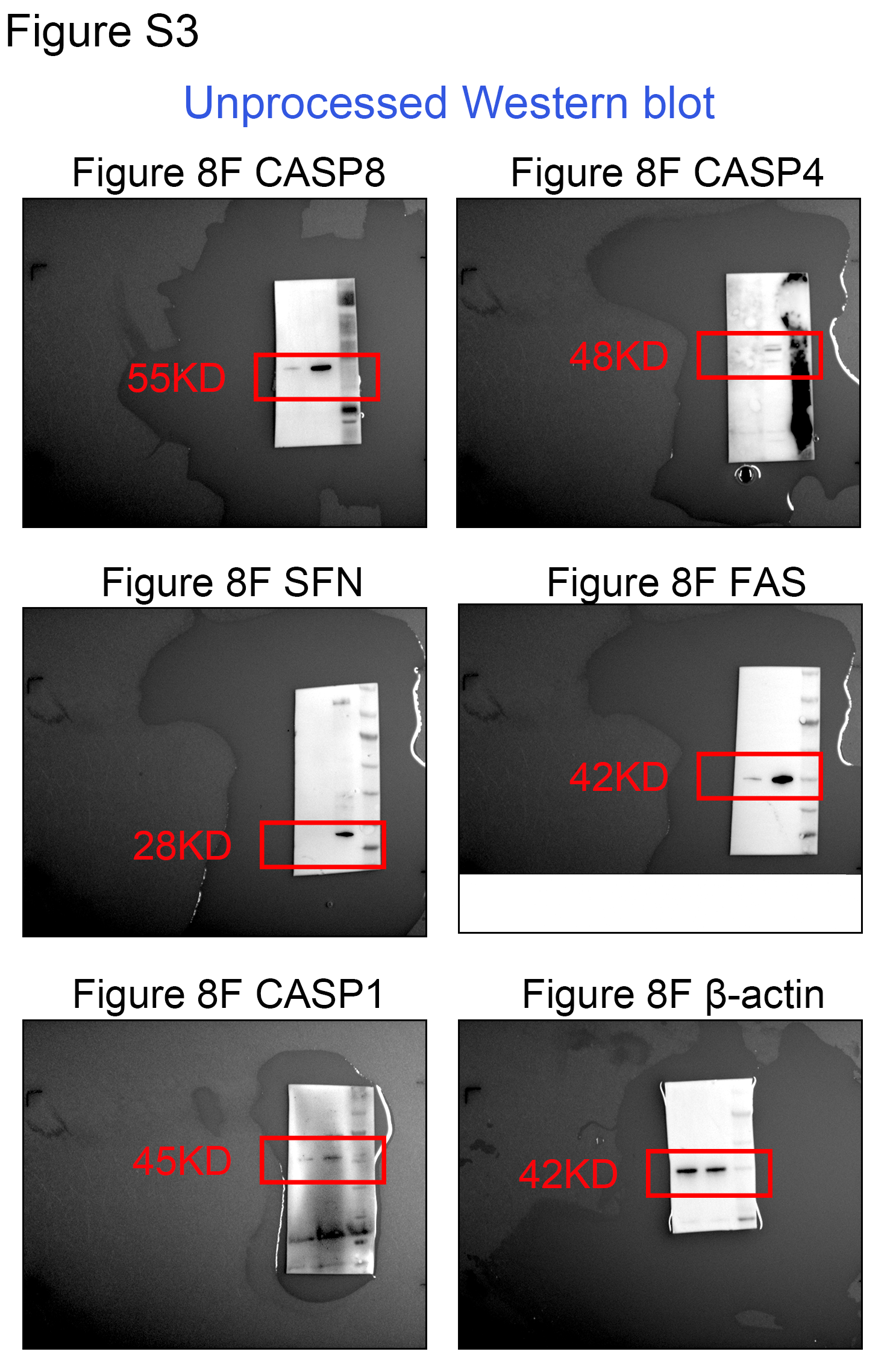

Supplement: Figure S3.tif [file IANN_A_2553930_SM3911.tif]

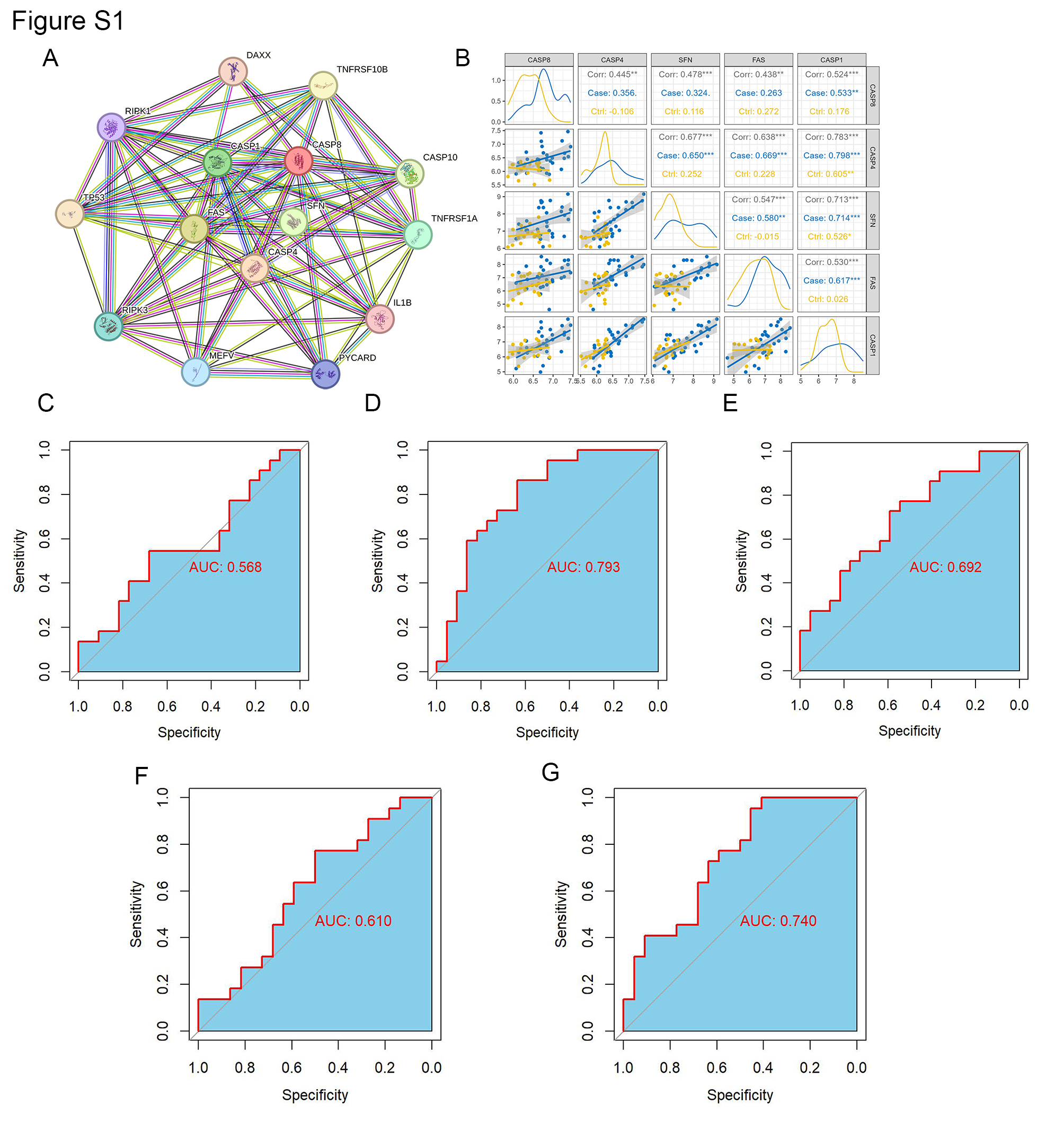

Supplement: Figure S1.tif [file IANN_A_2553930_SM3908.tif]

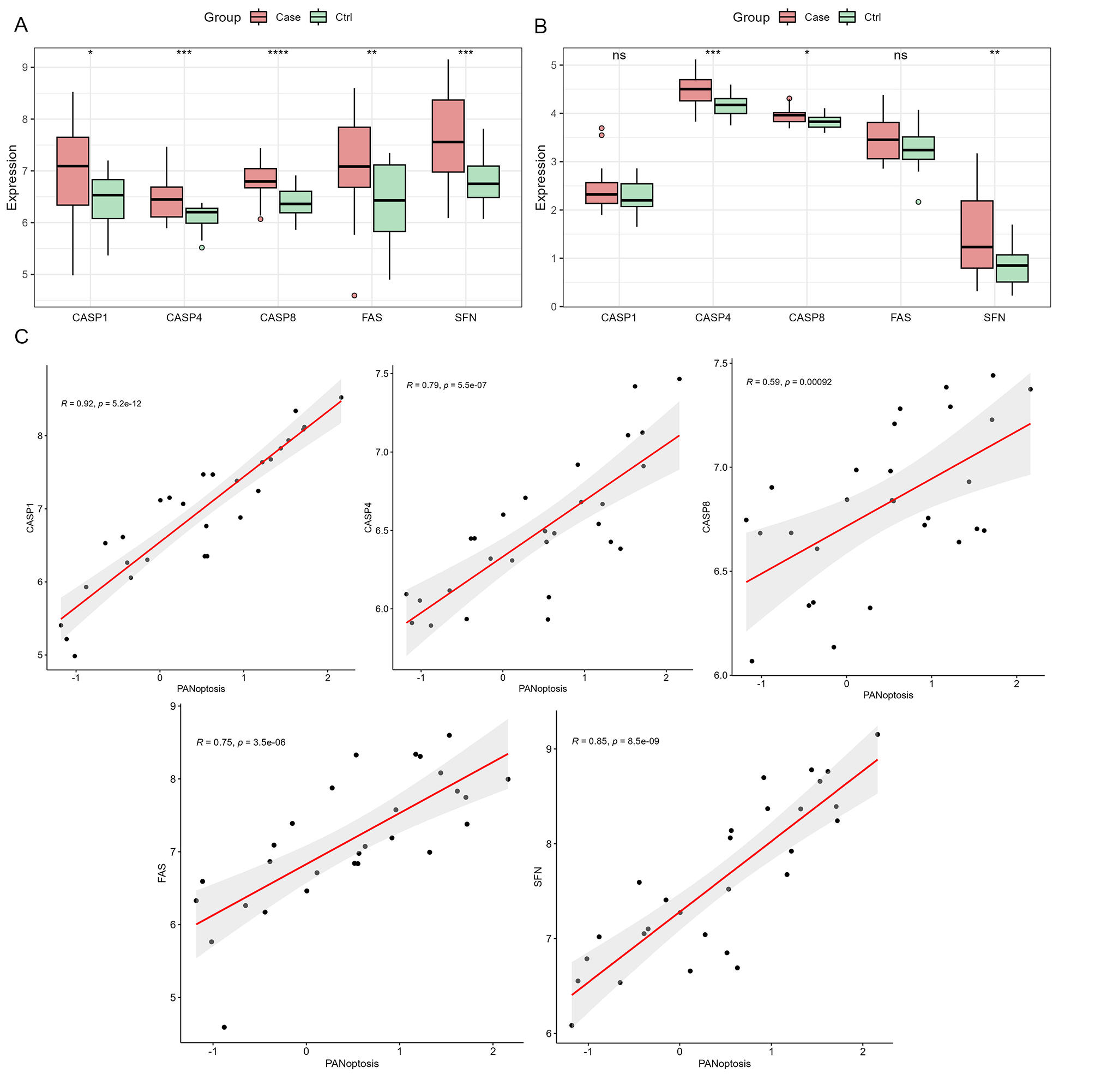

Supplement: Figure_S2 (1).tif [file IANN_A_2553930_SM3907.tif]
